# Use of transcranial magnetic stimulation in the treatment of nonfluent primary progressive aphasia: a case report

**DOI:** 10.1590/1980-5764-DN-2023-0021

**Published:** 2023-12-04

**Authors:** Natália Maria Lins Martins, Tathiana Baczynski, Larissa Sena, Romário de Macedo Espíndola, Natia Horato, Antonio Egidio Nardi, Valeska Marinho

**Affiliations:** 1 Universidade Federal do Rio de Janeiro, Instituto de Psiquiatria, Centro para Doença de Alzheimer, Rio de Janeiro RJ, Brazil.; 2 Universidade Federal do Rio de Janeiro, Instituto de Psiquiatria, Laboratório de Pânico e Respiração, Rio de Janeiro RJ, Brazil.

**Keywords:** Primary Progressive Aphasias, Dorsolateral Prefrontal Cortex, Transcranial Magnetic Stimulation, Afasia Primária Progressiva, Córtex Pré-Frontal Dorsolateral, Estimulação Magnética Transcraniana

## Abstract

Primary progressive aphasia comprises a group of neurodegenerative diseases characterized by progressive speech and language dysfunction. Neuroimaging (structural and functional), biomarkers, and neuropsychological assessments allow for early diagnosis. However, there is no pharmacological treatment for the disease. Speech and language therapy is the main rehabilitation strategy. In this case report, we describe a female patient diagnosed with nonfluent primary progressive aphasia who underwent sessions of high-frequency transcranial magnetic stimulation in the left dorsolateral prefrontal cortex and showed improvement in depression scores, naming tasks in oral and written speech, and comprehension tasks in oral and written discourse.

## RESUMO

As afasias progressivas primárias (APP) representam um grupo de doenças neurodegenerativas caracterizadas por disfunção progressiva da fala e da linguagem. A neuroimagem (estrutural e funcional), os biomarcadores e as avaliações neuropsicológicas permitem o diagnóstico precoce. No entanto, não há tratamento farmacológico para a doença. A terapia fonoaudiológica é a principal estratégia de reabilitação. Neste relato de caso, descrevemos uma paciente com diagnóstico de APP não fluente que foi submetida a sessões de estimulação magnética transcraniana de alta frequência no córtex pré-frontal dorsolateral esquerdo e apresentou melhora nos escores de depressão, nas tarefas de nomeação da fala oral e escrita e nas tarefas de compreensão da fala oral e escrita.

## INTRODUCTION

Primary progressive aphasia (PPA) comprises a group of neurodegenerative diseases characterized by progressive speech and language dysfunction with preserved functionality until advanced stages^1,2^. Three subtypes differing in clinical presentation, language dysfunction, and anatomopathological basis have been identified and described, namely:

- Nonfluent PPA (NFPPA);
- Semantic or fluent PPA (PPAS); and
- Logopenic PPA^3^.

Despite advances in terms of diagnosis and early detection of the disease, there is a lack of specific treatment for this condition. Pharmacological treatment for neuropsychiatric symptoms and speech and language therapy are currently the standard of care for PPA^4,5^. Recently, some reports have described a positive effect of repetitive transcranial magnetic stimulation (rTMS) on language and behavioral issues in PPA^6,7^, both over the left dorsolateral prefrontal cortex (DLPFC)^7^ and over the right and left DLPFC^6^. The rTMS is a safe method that can be used to promote a focal stimulus and generate an electromagnetic field through good shots using a coil. This method has a low risk of side effects, such as scalp pain, headache, and, rarely, epilepsy; furthermore, the side effects have been reported to be tolerable^8^.

According to the previously reported data^6,7^, we hypothesized that rTMS over the left DLPFC could improve language parameters, and considering the well-known effects of rTMS on depressive symptoms, a secondary beneficial effect on behavioral symptoms was supposed.

We describe a case report of a middle-aged woman diagnosed with NFPPA with a pathogenic mutation identified in the LRRK2 gene who underwent rTMS sections with positive results both in language parameters and behavioral symptoms.

## CASE REPORT

A 55-year-old female, divorced, who worked as a university teacher sought medical care due to “difficulty communicating”. Her symptoms began during the previous four years with progressive difficulties in sentence construction, and she reported “finding it hard to complete a sentence”. After the initial symptoms, language deficits compelled her to quit her job. During the few months before the presentation, her language issues became exacerbated, and she required assistance with writing. Her behavior remained unaltered, but she began to experience difficulty carrying out day-to-day activities due to her language deficits.

At the first consultation, in addition to language problems, the patient also reported a depressed mood for the past three months, and she had been prescribed mirtazapine at a dosage of 30 mg/day. She did not have any other psychiatric or neurological disorders, and her clinical and neurological evaluations were normal. The patient had been receiving language therapy for more than six months. Biomarkers and complementary tests needed for a precise diagnosis of PPA were requested and are described below.

**Figure 1:**
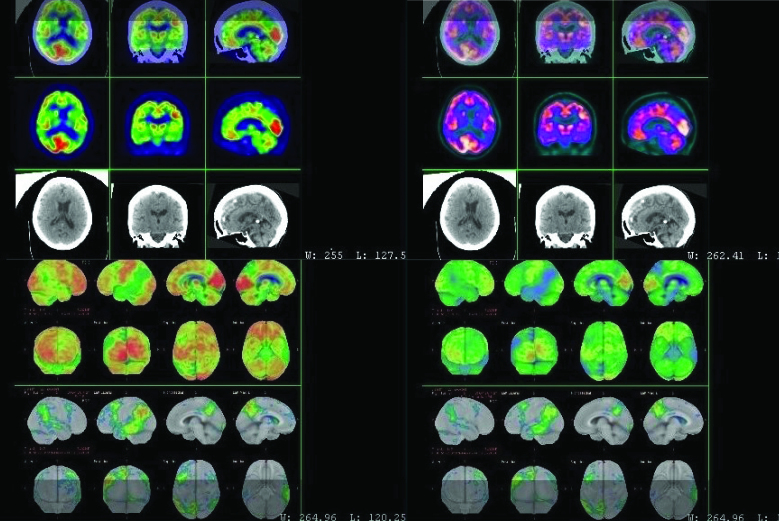
Fluorodeoxyglucose-positron emission tomography (FDG-PET) sequences showing moderate/marked reduction in glycolytic metabolism in the lateral regions of the left temporal lobes and the lower portions of the parietal lobes.

The neuropsychological evaluation at the moment of her first consultation revealed grammatical, praxis, and verbal fluency deficits, keeping naming and word comprehension ability preserved. The fluorodeoxyglucose-positron emission tomography (FDG-PET) revealed a moderate/marked reduction in glycolytic metabolism in the lateral regions of the temporal lobes and the lower portions of the parietal lobes; there was an asymmetrical pattern due to greater left-side involvement, especially in the lateral portion of the prefrontal cortex, in the posteromedial portions of the parietal lobes and in the posterior gyrus of the cingulate cortex (Figure 1).

**Figure 2:**
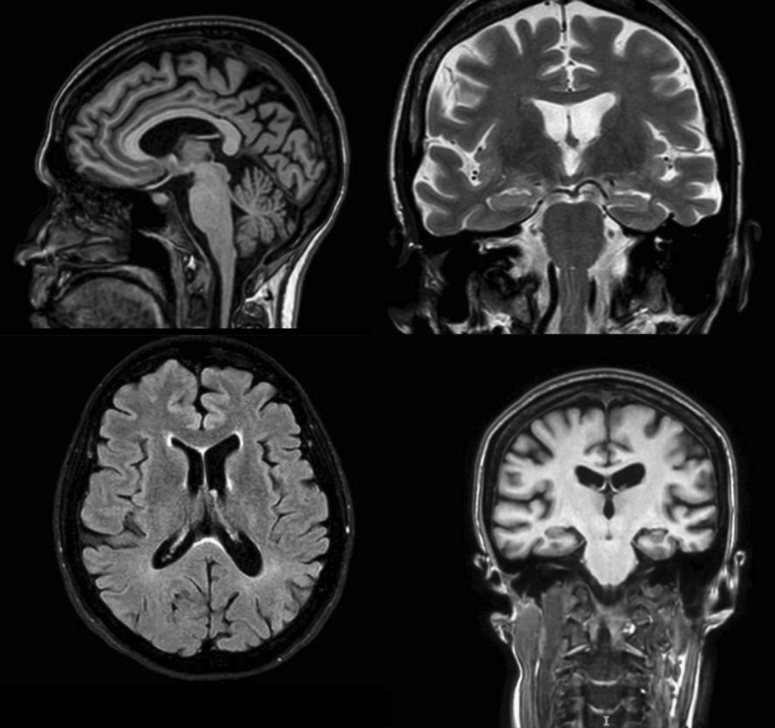
Magnetic Resonance Images of rare foci with signal on T2 fluid-attenuated inversion recovery, suggestive of minimal gliosis/ischemia due to degenerative microangiopathy, scattered in the periventricular white matter and frontal and parietal lobes (Fasekas 1). Hippocampus of normal volume and signal intensity (MTA:1). Cortical grooves, fissures and basal cisterns accentuated.

Magnetic resonance imaging (MRI) of the skull revealed gliosis due to atherosclerotic microangiopathy and prominent cisterns, fissures, and cortical grooves; additionally, compensatory ectasia of the lateral ventricles was observed (Figure 2).

Cerebrospinal fluid (CSF) analysis showed an increase in total tau (t-tau) levels (496,9 pg/ml/I.R.: <300 pg/ml (21–50 years); <450 pg/ml (51–70 years); <500 pg/ml (71–93 years) and phosphorylated tau (p-tau) protein (107,2 pg/ml/I.R.: <61 pg/ml), a decrease in beta-amyloid protein Aβ1-42 levels (338,7 pg/ml/I.R.: >500 pg/ml), and a decrease in Aβ1-40 levels (3919 pg/ml/I.R.: 7755–16715 pg/ml). Nonetheless, the Aβ1-42/Aβ1-40 ratio (0,086 pg/ml/I.R.: >0,069 pg/ml) was within the normal range. A genetic panel was performed for several genes, including some already related to neurodegenerative diseases and a pathogenic variant was found: c.6055G>A (p. Gly2019Ser). This variant was identified in leucine-rich repeat kinase 2 (LRRK2). Pathogenic mutations in the LRRK2 gene are associated with Parkinson’s disease, and some reports describe preliminary evidence correlating with Corticobasal Syndrome (CBS) and PPA^9,10^.

## METHODS

The project was approved by the IPUB/UFRJ (*Instituto de Psiquiatria da Universidade Federal do Rio de Janeiro*) Ethics Committee, CAAE: 61534322.5.0000.5263. The participant was informed about the procedure and signed the informed consent form.

The rTMS protocol consisted of one daily 15-minute session across three to five consecutive days per week for six consecutive weeks (a total of 28 sessions). The patient kept her daily routine as usual including conventional speech and language therapy. She had submitted to this therapy, focusing on discourse and language production, of one-hour duration, every week for the last six months. The patient underwent 28 high-frequency rTMS (HF-rTMS) sessions to the left DLPFC. The stimulation intensity was gradually increased, to reduce any discomfort associated with the procedure, with no change in the motor threshold (MT) over the weeks of stimulation. Thus, for the first four sessions, an intensity of 100% of the calculated MT was used; for the next four sessions, the intensity was 110% of the MT; and, for the last 20 sessions, the intensity was 120% of the MT, which was kept stable until the last rTMS procedure. The following parameters were also used: 10 Hz frequency; 5s series duration; 25s interval duration; and 15-min total session duration. The rTMS protocol, including stimulus duration and number of sessions, was based on the approved rTMS protocols by the Brazilian Federal Council of Medicine in 2012^11^. No changes in prescription patterns and/or language therapies were allowed during the procedure. Eleven days before and four days after the last rTMS, the patient completed depression symptom scales (Cornell Scale and the Geriatric Depression Scale – GDS), global cognitive measures (Alzheimer’s Disease Assessment Scale — Cognitive Subscale — ADAS-Cog and Mini-Mental State Examination — MMSE), and a specific language evaluation conducted by a speech therapist (Montreal-Toulouse Language Assessment Battery — MTL)^12^.

A table containing the results of the MRL-BR, Brazilian version (MTL-Brazil) pre- and pos-rTMS procedure is available as Supplementary Material.

## RESULTS

The patient showed reductions in depression symptoms on both the Cornell Scale (7 points and 2 points at pre- and post-rTMS, respectively) and the GDS (12 points and 6 points at pre- and post-rTMS, respectively). The patient also improved in global cognitive function on the MMSE (18/30 and 20/30 at pre- and post-rTMS, respectively) and the ADAS-Cog (42,7 and 35,7 at pre- and post-rTMS, respectively).

**Figure 3:**
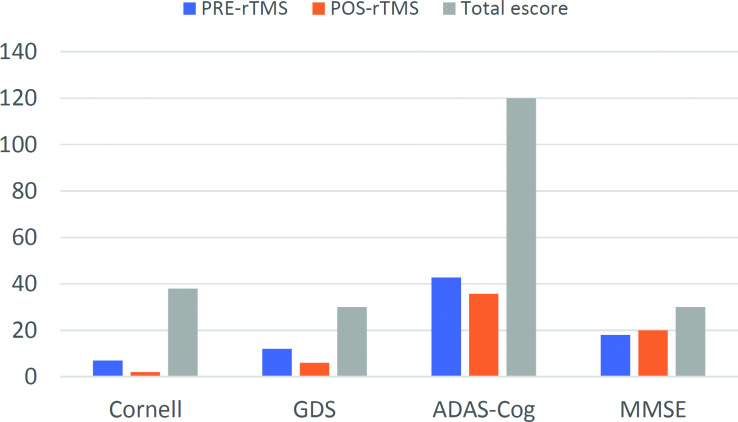
Pre- and pos- repetitive transcranial magnetic stimulation and total score.

Abbreviations: rTMS, repetitive transcranial magnetic stimulation; GDS, Geriatric Depression Scale; ADAS, Alzheimer’s Disease Assessment Scale; MMSE, Mini-Mental State Examination.

Furthermore, the patient presented improvements in the following language parameters as assessed by the MTL-Brazil: oral and written comprehension of sentences, copy, writing under dictation, sentence repetition, read-aloud sentences, naming abilities for nouns and verbs, manipulation of objects under verbal order, and written naming for nouns. The patient showed decreases in oral narrative speech, semantic verbal fluency, phonological/orthographic verbal fluency, written nomination for verbs, reading numbers, written narrative speech number of words, and written text comprehension. No difference was found in automatic speech for form and content, oral comprehension for nouns, number of information units (IU), scenes, written words comprehension, word repetition and reading, nonverbal praxis, recognition of body parts and notions of right and left, listening comprehension, written narrative speech and text comprehension, and numerical calculation (Figure 3 and Table 1).

**Table 1:** Montreal-Toulouse Language Assessment Battery, Brazilian version results, pre- and pos- repetitive transcranial magnetic stimulation.

	PRE-rTMS	POS-rTMS	Total score
Direct interview	20	20	26
Automatic speech (form)	6	6	6
Automatic speech (content)	6	6	6
Oral comprehension	12	16	19
Words	5	5	5
Sentences	7	11	14
Oral narrative speech (nº words)	59	41	–
Total IU	3	3	10
Total scenes	0	0	3
Written comprehension	10	11	13
Words	5	5	5
Sentences	5	6	8
Copy	6	8	8
Writing under dictation	10	14	22
Repetition	10	24	33
Words	11	11	11
Sentences	7	13	22
Read aloud	32	33	33
Words	12	12	12
Sentences	20	21	21
Semantic verbal fluency	9	8	–
Non-verbal praxis	24	24	24
Oral nomination	26	30	30
Nouns	22	24	24
Verbs	4	6	6
Manipulation of objects under verbal order	11	14	16
Phonological/orthographic verbal fluency	8	7	–
Recognition of body parts and notions of R/L	8	8	8
Body segments	4	4	4
R/L perceptions	4	4	4
Written nomination	21	28	30
Nouns	15	21	24
Verbs	6	4	6
Listening comprehension of the text	2	2	9
Numbers dictation	5	5	6
Reading numbers	6	5	6
Written narrative speech (nº words)	37	22	–
IU Total	3	3	10
Scene score	0	0	3
Written text comprehension	4	0	9
Numerical calculation	0	0	12
Mental	0	0	6
Numerical	0	0	6

Abbreviations: rTMS, repetitive transcranial magnetic stimulation; IU, information units; R/L, right/left.

The patient was also asked to rate her condition after the procedure and report an improvement in her daily life activities.

## DISCUSSION

This case study revealed the beneficial effects of rTMS applied to the left DLPC in a 55-year-old woman with NFPPA related to LRRK2 pathogenic mutations. The results indicated reductions in depressive symptoms and improvements in both global cognitive function and language parameters.

Considering the scarcity of therapeutic approaches that can modify or stop the evolutionary course of PPA^13^, and based on the promising results of rTMS in other neuropsychiatric disorders^14^, the present case report sheds light on this noninvasive neuromodulation method as a treatment option for PPA. The best-established indication for rTMS is depression treatment via stimulation of the left dorsolateral prefrontal cortex^15^. Our report is in line with these results since an improvement in depressive symptoms was observed. The scores on the GDS and Cornell Scale were lower after the procedure. Global cognitive function and language parameters were also improved as assessed by the ADAS-Cog, MMSE, and MTL-Brazil. The positive effects on cognition could be a direct effect of rTMS or a secondary effect due to mood benefits generated by the procedure. However, benefits in cognition and language production were measured before and after stimulation and resulted in benefits for global cognition, action, and object naming abilities and language skills such as comprehension of sentences, copying, and writing under dictation. These results corroborate previous studies that reported direct cognitive effects with rTMS in PPA^6,7,16,17^. Finocchiaro et al. described the first clinical case report of PPA with HF-rTMS applied to the left prefrontal cortex. The patient was evaluated with a language battery of tests before and after treatment, and the results showed significant improvements in vocal production after rTMS^16^.

In 2012, Cotelli et al. conducted a clinical trial of ten NFPPA subjects who underwent rTMS applied to the DLPFC. The results pointed to effects on action naming performance for patients who received stimulation. No facilitating effect of rTMS object naming was observed^6^. Margolis et al. carried out a single-blind study including six NFPPA subjects submitted to HF-rTMS sham and rTMS applied to the right or left DLPFC. The most consistent result was a statistically significant improvement in action naming post-rTMS applied to the left DLPFC but not to the right DLPFC. There were no benefits for object naming after right or left DLPFC rTMS stimulation. There was an improvement in global cognitive performance in both groups as measured by the Montreal Cognitive Assessment (MoCA). The results pointed to benefits in naming actions and global cognitive improvement for rTMS to the left DLPFC, suggesting that stimulation to the left DLPFC may be more beneficial than to the right DLPFC^7^.

The first randomized placebo-controlled study included 20 nonfluent and semantic PPA patients enrolled in active- versus control-site rTMS in a crossover design. Despite being a more robust study design, the stimulation protocol used was heterogeneous, associated with multiple foci, in addition to excitatory or inhibitory stimuli. This study found improvement in spontaneous speech as a primary outcome and other positive results in object naming and syllable tasks for subjects in the active group^17^.

PPA is a rare condition with different clinical subtypes, language-related symptoms, and various associated anatomopathologies, with no pharmacological treatment currently available. Recent data suggest the benefits of speech therapy for trained words^13^. The present case study revealed the positive effects of supplementing speech therapy with rTMS as a potential option to improve mood and cognitive function. Future randomized placebo-controlled studies with large sample sizes should be conducted to evaluate the potential of rTMS over the left DLPFC as an adjuvant to speech-language therapy in PPA.

A limitation of the present study include the case-report design describing rTMS benefits for a single NFPPA patient, which precludes generalization to other PPA patients and to other clinical PPA variants. A possible placebo effect cannot be ruled out, as the study lacks a control condition of neuromodulation. The single case report method prevents a comparator for other subjects, and as no information about disease progression was available, a comparator for the patient herself could not be made. Besides this lack of baseline measures, another possible limitation is the absence of functional neuroimages before and after the stimulus procedure that could have added neurobiological measures of efficacy. The patient remained on her usual treatment with speech and language therapy, so a possible beneficial effect of such behavioral intervention could not be ruled out. Further, evaluations were performed shortly after stimulation, and the duration of the effects was not examined. Thus, the present report has encouraging findings that are limited to the patient described herein. There is a need for more robust and longitudinal studies of rTMS on PPA patients.
